# Liquid Flow Meter by Fiber-Optic Sensing of Heat Propagation

**DOI:** 10.3390/s21020355

**Published:** 2021-01-07

**Authors:** Alin Jderu, Marcelo A. Soto, Marius Enachescu, Dominik Ziegler

**Affiliations:** 1 S.C. NanoPRO START MC S.R.L., Oltenitei, No. 388, District 4, 041337 Bucharest, Romania; alin.jderu@cssnt-upb.ro; 2 Center for Surface Science and Nanotechnology (CSSNT), University Politehnica Bucharest, 060042 Bucharest, Romania; marius.enachescu@cssnt-upb.ro; 3 Department of Electronic Engineering, Universidad Técnica Federico Santa María, 2390123 Valparaíso, Chile; marcelo.sotoh@usm.cl; 4 Academy of Romanian Scientists, 54 Splaiul Independentei, 050094 Bucharest, Romania

**Keywords:** distributed optical fiber sensing, optical frequency-domain reflectometry, flow rate monitoring, flow diagnostics

## Abstract

Monitoring fluid flow rates is imperative for a variety of industries including biomedical engineering, chemical engineering, the food industry, and the oil and gas industries. We propose a flow meter that, unlike turbine or pressure-based sensors, is not flow intrusive, requires zero maintenance, has low risk of clogging, and is compatible with harsh conditions. Using optical fiber sensing, we monitor the temperature distribution along a fluid conduit. Pulsed heat injection locally elevates the fluid’s temperature, and from the propagation velocity of the heat downstream, the fluid’s velocity is determined. The method is experimentally validated for water and ethanol using optical frequency-domain reflectometry (OFDR) with millimetric spatial resolution over a 1.2 m-long conduit. Results demonstrate that such sensing yields accurate data with a linear response. By changing the optical fiber interrogation to time-domain distributed sensing approaches, the proposed technique can be scaled to cover sensing ranges of several tens of kilometers. On the other extreme, miniaturization for instance by using integrated optical waveguides could potentially bring this flow monitoring technique to microfluidic systems or open future avenues for novel “lab-in-a-fiber” technologies with biomedical applications.

## 1. Introduction

Monitoring fluid flow rates accurately is key in many industries with processes such as drug delivery, food and beverage processing, biomedical engineering, chemical engineering, and pipeline monitoring in the oil and gas industry [1,2,3]. For the different kind of applications and length scales, there exist many optimized flow metering techniques. For flow rates lower than one milliliter per minute, thermal flow sensors based on micro-electromechanical systems are most widely used [4,5]. Most implementations use a central heating element and infer flow rates from the heat transport to two or more nearby thermometers [6]. Although they offer excellent performance, they are complex to manufacture, require calibration depending on the fluid, are prone to clogging, and are incompatible with harsh environments such as high temperature, corrosive fluids, or strong electromagnetic fields. Optical fiber-based flow sensors to date operate using optical fiber interferometry [7,8] or optical hot-wire anemometry [9,10]. Hot-wire anemometers estimate flow rates by measuring the heat losses from a heating element with a fiber temperature sensor, such as fiber Bragg gratings [9,10]. Unfortunately, the major drawback of anemometers is their inability to detect flow direction.

Distributed optical fiber sensing offers unique monitoring possibilities, such as enabling simultaneous multiple-point monitoring of strain and temperature with one single optical fiber [11,12]. With many specialized detection modes, distributed optical fiber sensing has found diverse applications in monitoring communications networks, structural health monitoring, shape sensing, pipeline and electrical transmission line monitoring, intrusion detection for perimeter security applications, and geo-hydrological monitoring [11,12,13,14]. Recently, we have reported flow monitoring using distributed temperature sensing with continuous heating [15]. In this Letter, a novel method to measure flow rates based on distributed temperature sensing and pulsed heating injection is proposed. We explore and discuss different methods to extract flow rates and effects of thermal diffusivity of the fluidic system. Rather than depending on a reliable absolute temperature measurement at one or a few locations along the conduit, the method infers flow velocity from the analysis of the heat wave propagation. This makes the method tolerant to external influences including temperature of the environment or fluid. The method uses optical frequency-domain reflectometry (OFDR) for temperature recordings with millimetric spatial resolution over a short fluidic conduit, and it can be applied with other distributed optical fiber sensing approaches, such as time-domain techniques, to enable the monitoring of fluid flows over several tens of kilometers.

## 2. Materials and Methods

### 2.1. Experimental Setup

**Figure 1:**
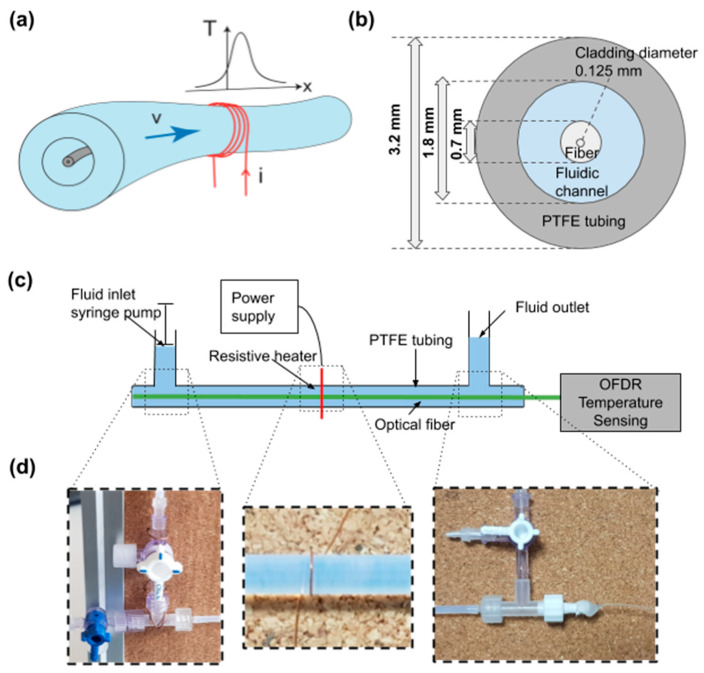
(**a**) Concept of flow monitoring using distributed optical fiber temperature sensing. A pulsed electric current (i) is applied to a copper coil wrapped around a fluidic conduit (red coil). Joule heating locally elevates the temperature (T) of the fluid propagating at a flow velocity (*v*). The optical fiber inserted in the fluidic conduit monitors the resulting temperature distribution T(x,t). (**b**) Transverse cross-section of the fluidic conduit with an inserted optical fiber that defines the remaining area as resulting annular flow channel. (**c**) Schematic of the experimental setup. A syringe pump controls the fluid flow and optical frequency-domain reflectometry (OFDR) is used for distributed temperature sensing. (**d**) Close-up photographs highlight the easy-to-build implementation. From left to right shown are the fluidic inlet connecting to a syringe pump, the single copper coil as heating element, and the use of a fluidic T-connector (Swivel Luer Locks) to couple the fiber straight into the tubing without bending and risk to apply high strains. A hot gun glue seals off the tube and prevents leakage. The extra manual valves at the inlet and outlet help to rinse the conduit or exchange liquids quickly without using the syringe pump. To minimize gravitational pressure effects that could lead to back pressure, a wider diameter syringe without a piston was added as a reservoir at the fluid outlet.

Figure 1a shows the experimental layout of the proposed method. An optical fiber is inserted into a polytetrafluoroethylene (PTFE) tubing around which a heating coil is wrapped. When an electric current is applied to the coil, Joule heating locally elevates the temperature of the fluid. Mass flow convectively spreads the heat downstream, and the optical fiber is interrogated by a commercially available OFDR system (ODiSI-B by Luna Technologies Inc., Roanoke, VA, USA) to monitor the resulting temperature profile T(x)  inside the PTFE tube with high spatial resolution. OFDR is a well-established method for measuring the temperature profile of an optical fiber. The method is based on swept wavelength interferometry, where the Rayleigh scatter from the fiber is mixed with a reference and detected. The output is Fourier-transformed to yield spatial information [16]. All recordings are performed at a rate of 4 Hz and contain 1800 data points along the 1.2 m long fiber. To control the flow, we use a syringe pump to install volumetric flow rates between 10 and 1000 µL/min. As indicated in Figure 1b, the inner radius of the PTFE tubing is a = 1.8 mm and the outer radius of the optical fiber is b = 0.7 mm. Considering that the optical fiber is placed inside the conduit, the remaining cross-sectional area of the annular channel is $A=\pi \left(a^{2}-b^{2}\right)\;=$ 2.16 mm^2^. Note that by placing the optical fiber inside the PTFE tubing, an efficient thermal transfer from the fluid to the optical fiber could be secured. This is indeed essential for a reliable sensing, especially for high flow rates. The only limitation of this approach is given by the transversal area of the conduit, which should be much higher than the fiber cross-section, as in the present case, to minimize invasive effects on the fluid flow. Placing the optical fiber externally to the conduit could result in a weaker thermal transfer to the optical fiber, thus eventually affecting the response of the sensor. The highest volumetric flow rate applied in this experiment is 1000 µL/min, which corresponds to an average scalar flow velocity of 7.7 mm/s. With a calculated Reynolds number of 9.6, we are asserting operation in the laminar flow regime [17]. Local heating of the fluid is achieved by passing an electric current of 1.5 A through a single loop of a 200 µm thick copper wire wrapped around the tubing (as shown in Figure 1d). Flow, heating, and temperature data collection are synchronized with a dedicated LabVIEW interface.

OFDR-based measurements require a reference distributed measurement at a known stable temperature condition; for this, calibration heating needs to be turned off, but the flow does not need to be stopped. Then, the actual temperature measurements are obtained with applied heating to monitor the fluid’s overheating temperature, thus giving a spatial sampling of 0.67 mm. The build-up of temperature during a continuous heating (up to *t* = 30 s) has been studied in great detail using finite element analysis and experiments [15]. Estimating flow rates based on continuous heating essentially requires monitoring the propagation of the temperature rising edge along the conduit, which is affected by the thermal diffusivity of the fluid and the entire system. For monitoring of flow rates, a pulsed heating approach is more practical and reliable. The displacement of heat between two pulses becomes straightforward to analyze without the additional heat influx.

### 2.2. Visualizing Heat Propagation

In the presence of a temperature gradient in a liquid medium, convection occurs. It causes the actual movement of molecules from a high-temperature region to a low-temperature region (diffusion). The transfer of energy takes place due to the bulk/macroscopic motion of fluids. In natural convection, the flow is caused due to buoyancy effects in the fluid, while in the case of forced convection, an external source (e.g., a fan or pump, etc.) causes the flow. Irrespective of the nature of convection, the heat flow rate equation can be expressed using Newton’s law of cooling. 

The transfer of heat away from a heater can be described using the convection–diffusion equation $\partial T/\;\partial t\;=\;\nabla \cdot \left(\alpha \nabla T\;\right)\;-\nabla \cdot \left(\vec{V}T\right)\;+\;H\;\left(x,t\right)\;+µ\left(x,t\right)$, where T is temperature, α is the fluid’s thermal diffusivity, $\vec{V}$ is the fluid’s velocity vector at any point, H(x,t) is any change in temperature forced upon the system, and µ(x,t) are the thermal losses to the environment [18].

**Figure 2:**
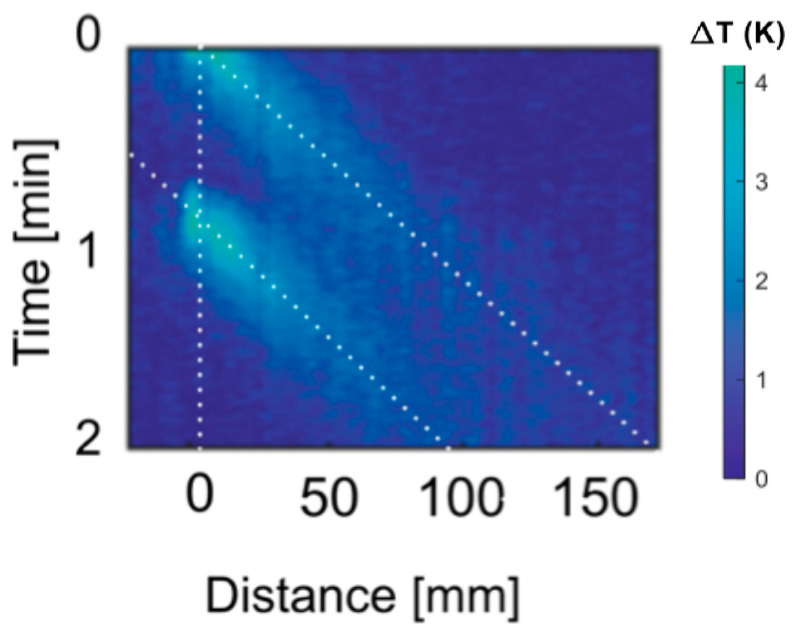
Two-dimensional (2D) color map image of the temperature increase against time and distance when using two consecutive heating pulses. The slope of the dotted lines corresponds to the inverse of the flow velocity in mm/min. The color bar indicates the measured temperature increase (in Kelvin). For the set flow rate of 200 µL/min and the given cross-sectional area of 2.16 mm^2^, an average flow velocity of 93 mm/min is expected.

The response to the convection–diffusion equation is visualized by our experiment where a constantly widening and shifting temperature peak travels downstream. When convection is the dominant method of heat transfer, the peak location can be tracked easily to derive the flow velocity. The relative dominance of convection over diffusion is described by the Peclet number $Pe\;=\;Lv\;\alpha^{-1}$, where L is the characteristic length, v is the flow velocity, and α is the thermal diffusivity of the fluid. For a Peclet number greater than one, the system is dominated by convection [18,19]. For low flow rates and short distances, diffusion is the dominant method of heat transfer, and heat will dissipate before it moves over a significant distance. With continuous heating, flow rates down to 5 µL/min could be resolved [15]; however, a non-linear response to flow rates was observed, and finding quantitative results would require precise calibration. However, in the here-presented measurements obtained with pulsed heating, the flow measurement is very visual, and the results are simple to extract. Figure 2 shows the measured convective heat flow as it propagates downstream in a 2D color map of the temperature increase against distance and time. For a set flow rate of 200 µL/min and the given geometry, we find an average flow velocity of 93 mm/min. Following the diagonal white dotted lines in the figure, this flow rate can be readily visualized and confirmed to match with the actual applied flow rate. To secure that the heat from the two pulses remains easily discernible, a pulse interval of 1 min is chosen for this flow rate. However, this interval depends on the flow rate range that is targeted with the sensor; thus, for fast flow rates, it would be advantageous to have a shorter pulse repetition interval.

### 2.3. Peak Detection vs. “Center of Heat” Detection

**Figure 3:**
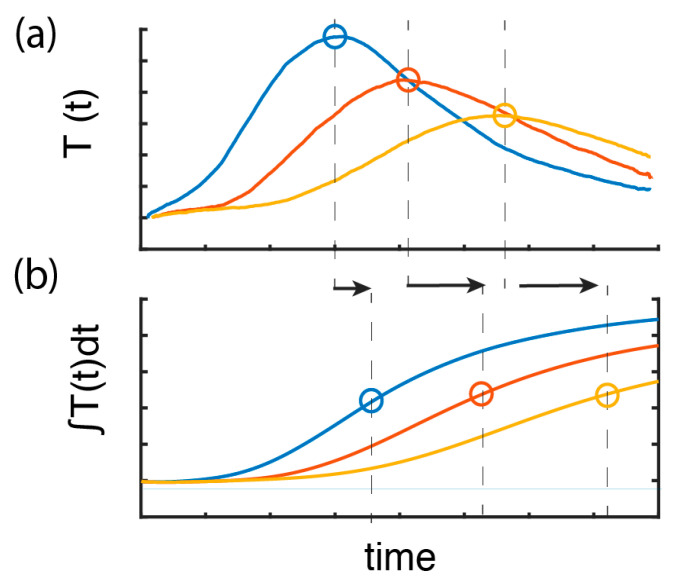
Description of two methods to estimate flow velocity. (**a**) Temperature profiles T(x,t) measured at three different fiber locations, indicating that the peak temperature can be tracked over the time–distance domain to extract the flow velocity. Convective heat transport distorts the symmetry of the heat pulse propagating downstream; therefore, this method results in a significant underestimation of the flow rates. (**b**) The integral of the temperature profile over time for three different fiber positions, from which the “center of heat” is obtained as the time when the integral of T(x,t) reaches half the maximum value for each position. Tracking this center point gives information of the propagation of the centroid of the thermal energy pulse. The black arrows highlight the difference between the identified times to calculate flow rates.

Measurements of the heat propagation, as shown in Figure 2, must be properly processed to extract the flow velocity from the slope (or inverse slope) visualized in the data. Different strategies to analyze the heating temperature propagation can be followed to extract the velocity. Figure 3 illustrates the two strategies that are implemented in this work. A simple and straightforward method to extract the flow speed is to detect the peak of the temperature profile and follow its propagation in time and distance domain. This is exhibited in Figure 3a, which shows the temperature profile T(x,t) measured as a function of time at three different fiber locations. Tracking the propagation of the peak, a first approximation of the flow velocity can be obtained. However, as convective heat transport results in asymmetric profiles, as clearly observed in Figure 3a, the peak location of the heat pulse does not identify the center of a heated fluid. Under convective heat transport, the peak position shifts over time and distance, thus leading to an underestimation of the actual flow (note that the peak is located closer to the left edge of the heat pulse, thus explaining this underestimation). In analogy to the center of mass, we define as “center of heat” the time at which the integral of T(t) reaches half the maximum value for each position. Tracking the “center of heat” gives more precise estimations of the actual flow velocities, and hence of the flow rates than tracking the peak. For each location, we find the “center of heat” and use linear fitting to extract the velocity.

## 3. Results

### 3.1. Recordings of Heat Propagation for Water and Ethanol

**Figure 4:**
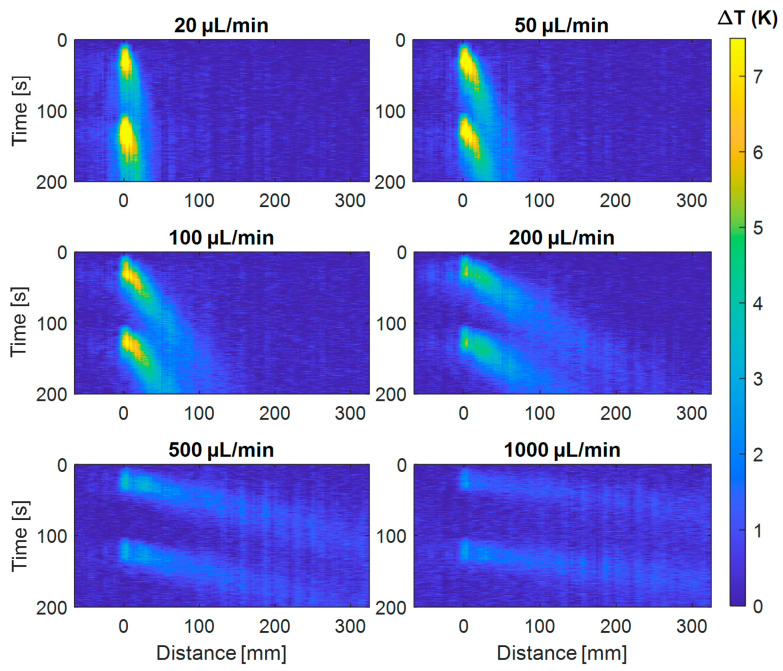
Propagation of the heating pulses induced in water at a fixed fiber position (indicated at a distance of 0 mm) as a function of time and distance for different flow rates. Two heating pulses are injected during 30 s and separated by a 70 s waiting period. The convective heat transport by the flow is clearly observable for the different flows. The color bar indicates the measured temperature increase ∆T (in Kelvin) of the water, which for low flow rates reaches up to ≈7 K above room temperature.

**Figure 5:**
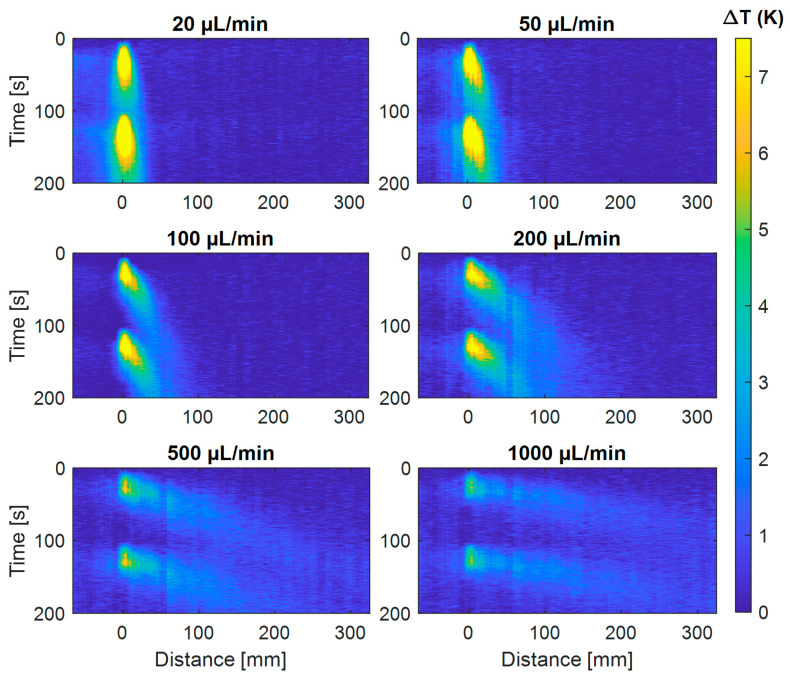
Propagation of the heating pulses induced in ethanol at a fixed fiber position (indicated at a distance of 0 mm) as a function of time and distance for different flow rates. Two heating pulses are injected during 30 s and separated by a 70 s waiting period. The convective heat transport by the flow is clearly observable for the different flows. The color bar indicates the measured temperature increase ∆T (in Kelvin) of the ethanol, which for low flow rates reaches up to ≈7 K above room temperature.

Using the OFDR system, the propagation of the induced heat pulse is monitored in the time and distance domains. Two heating pulses with a duration 30 s are applied to the fluid. The second pulse starts 70 s following the first. Then, the experiment is repeated at different flow rates ranging from 10 to 1000 µL/min. Figure 4 and Figure 5 show the temperature profile over the distance–time domain recorded for the different flow rates, for water and ethanol, respectively. Flows are being kept constant throughout each recording.

### 3.2. Estimating Measured Flow Rates

**Figure 6:**
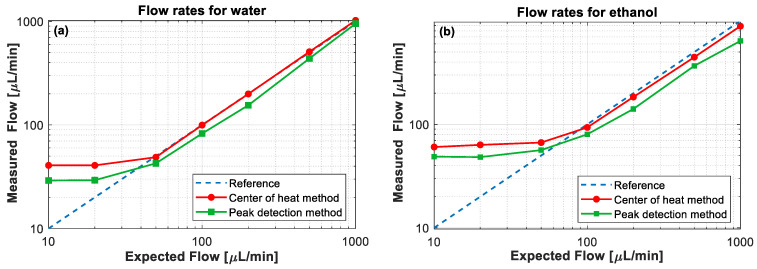
Volumetric flow rates deduced by both the peak detection and the center of heat methods described in Section 2.3 for (**a**) water and (**b**) ethanol. The blue dashed lines show the expected volumetric flow rates, while the red and green curves indicate the measured flow rates obtained by the center of heat and peak detection methods, respectively.

Using the peak and “center of heat” detection methods as described in Section 2.3 over the measurements depicted in Section 3.1, the flow velocity of water and ethanol has been estimated for the different flow rates. Making use of the cross-sectional area of the annular channel A = 2.16 mm^2^ and the estimated flow velocity v, the volumetric flow rate in volume units per minute can be obtained as F=60 vA for each case. Figure 6 shows the volumetric flow rates estimated from the proposed method as a function of the actually injected (expected) flow rates in the conduit. Results point out that when using the “center of heat” method, the flow rate matches very well with the expected values. The data for water are shown in Figure 6a. For flow rates above 50 μL/min, we find flow rates with an error of less than 1%. Note that there is calibration required in the entire system. The peak detection method (green curves) results in a substantial underestimation of the flow rates by about 20%. As described previously, this is due to the asymmetric profile from convective heat transport. The peak temperature position travels at a lower speed compared to the centroid of the thermal energy pulse. The data for ethanol is shown in Figure 6b. Here, we observe a 9.1% lower flow rate than expected even for the “center of heat” method. While the exact cause has not been identified yet, we believe that swelling of the polyimide coating of the fiber could play a critical role. Ethanol absorbed into the polymer matrix does not move freely with the flow in the channel, yet it affects the temperature measurement.

Flow rate detection limits are approximately 70 μL/min for ethanol and 40 μL/min for water. At low flow rates, two factors contribute to this limit: diffusion becomes the dominant method of heat transfer, causing broadening of the heat pulse and low displacement of the heat results in limits by the quantization of the time–distance domain of the measurement. Increasing the spatial resolution and sampling rate of the sensor, which is indeed very feasible for an OFDR system, could lead to a reduction of the lower limit of the flow rate that can be measured by the system. On the other hand, the maximum flow rate that can be measured depends on the thermal losses and diffusivity of the fluid and the system as a whole. These effects can be compensated by changing the heating temperature. In principle, to facilitate the recording of higher flow rates, it would be advantageous to use stronger heat injection; however, this may be limited by the type of material that the conduit is made of. In our current experiment, using PTFE tubing, we observed melting of the tubing for electric currents above 2A (note that the melting point of PTFE is 327 °C). We assume that with an increase in the heating temperature, the system could measure higher flow rates with detectable overheating temperature distributed over the entire length of the distributed sensor. Ongoing work focuses on improving data processing to extend the measurement range of the low Peclet number regime, where diffusion is the predominant method of heat transfer.

## 4. Discussion

We have performed pulsed heating experiments to demonstrate a simple method to process data and extract flow rates from analysis of the temperature distribution along the fluidic path. The optical fiber-based sensing approach is both a robust and easy-to-use solution, being also resilient to harsh conditions including high temperature, high pressure, corrosive media, and strong electromagnetic environments. No change in performance was observed even after several months of experiments with the fiber immersed in water, demonstrating that the system is maintenance free. Moreover, the sensor can be installed without being required to interrupt the conduit. The fluidic T-connector in the experiment (see Figure 1) is used to avoid sharp bends of the silica optical fiber in our specific implementation. The optical fiber can normally be inserted either from the fluidic inlet or outlet side without interruption of the conduit. In a real scenario, this is a feasible common solution, in which the only physical limitation is imposed on the bending radius of the silica fiber, which must be larger than a few cm. However, this is the same limitation for any optical fiber sensor. Compared to the prior method reported [15], the addition of a local pulsed heat source provides a linear response, offering also the possibility of discerning the origins of the heat distribution such as convection and diffusion. Typical methods that use only two or few temperature sensing locations cannot provide this information.

An optical fiber with one or even multiple heating elements can simply be retrofit to existing fluidic conduits. At each heater location, the conduit turns into a quasi-distributed flow meter that is read out with a single remotely located distributed fiber optical sensing interrogator. The high spatial resolution of OFDR interrogation provides accurate monitoring of the propagation of the heating pulses along the conduit, so that a precise time–distance map of the heating pulse could be demonstrated. This approach permits the distributed monitoring of the fluid flow at each location along the conduit, including the potential detection of leaks and turbulence effects, which cannot be obtained in a distributed manner by other existing approaches. Furthermore, synchronization of heating and data collection is not mandatory, because the heating can easily be identified in the temperature data. In addition to flow metering, the knowledge about temperature itself is useful to monitor exo- or endothermic reactions that occur for instance when mixing reacting fluids. Monitoring the temperature along fluidic conduits can improve the quality control of processes in the food industry, pharmaceutical, or chemical industries.

To improve the smallest detectable flow rate, ultra-small conduits in close proximity with the optical core could be realized when using the hollow channels of hollow photonic crystal fibers as fluidic conduits. With the reduction of the cross-sectional area down to a few µm^2^, we expect the detection limit could be reduced by three orders of magnitude. However, fluidic handling and interfacing with the micrometric channels would be challenging. Pressure-driven flow would become increasingly difficult the finer the conduits. On a larger length scale, optical fibers could be integrated into millimeter or centimeter-wide tubing. Ideally, the fiber would be integrated already when manufacturing the tubing in a polymer-extrusion process. In addition to the capability of flow monitoring, other functionalities such as strain or temperature recordings can provide useful information about the state of a fluid inside the tubing. Leak detection or monitoring of thermal effects by chemical reactions that are beyond the scope of this contribution would readily become available.

## 5. Conclusions

Using OFDR-distributed temperature sensing, we demonstrated a straightforward method to extract flow rates from analysis of the heat distribution inside a fluidic conduit. OFDR detection turns an optical fiber into thousands of independent temperature sensors. Hence, it enables the analysis of a distributed temperature distribution rather than a single localized temperature measurement. We have discussed the estimation of flow velocities using peak detection and a more sophisticated “center of heat” detection method, which identifies the center of the heat distribution profile and results in more accurate flow rate measurements. On the other hand, tracking the peak of the heat distribution reproducibly underestimates the flow rate. More advanced processing approaches can potentially push the detection limits even further and provide more reliable measurements at low flow rates when thermal diffusion dominates.

## Data Availability

Not applicable.
